# Identification of small molecule inhibitors of Ebola virus by use of a biologically contained virus system

**DOI:** 10.1016/j.virusres.2026.199823

**Published:** 2026-10-25

**Authors:** Meareg G. Amare, Peter J. Halfmann

**Affiliations:** Influenza Research Institute, Department of Pathobiological Sciences, School of Veterinary Medicine, University of Wisconsin-Madison, Madison, WI, 53706, USA

**Keywords:** Ebola virus, Filoviruses, Antivirals, Entry, Replication, Transcription

## Abstract

Ebola virus disease is a severe illness with high mortality for which limited therapeutic options are available. Identifying new antiviral drugs that target viral proteins is essential for improving prevention and treatment strategies. In this study, we screened a library of 2620 small molecules enriched for reported inhibitory activity against cellular and viral helicases to identify phenotypic inhibitors of a biologically contained virus, Ebola∆VP30 virus, in Vero cells stably expressing VP30 protein. Seven compounds significantly reduced virus titers at least 100-fold with minimal cytotoxicity and demonstrated low IC_50_ values. Two of these compounds completely inhibited EbolaΔVP30 virus infection, interfering with either virus entry or replication. Although these preliminary screening hits require further optimization and in vivo validation, their identification opens new avenues for anti-Ebola virus drug discovery.

## Highlights

• Biologically contained Ebola virus enabled BSL-2 antiviral screening.
• A library of 2620 putative helicase inhibitors was evaluated.
• Eleven compounds inhibited Ebola virus with low cytotoxicity.
• Hit compounds blocked virus entry or replication.
• New chemical scaffolds were identified for filovirus drug discovery.

## 1 Introduction

Ebola virus (formerly *Zaire ebolavirus*), a member of the genus *Orthoebolavirus* within the family *Filoviridae*, is a highly pathogenic virus that causes Ebola virus disease (EVD). Since its discovery in 1976, Ebola virus has caused numerous sporadic outbreaks, most notably the 2013–2016 West African epidemic, which resulted in over 28,000 infections and >11,000 deaths. The genus *Orthoebolavirus* includes six species: Ebola virus, Sudan virus, Bundibugyo virus, Tai Forest virus, Bombali virus, and Reston virus (Baseler et al., 2017).

Ebola virus is a non-segmented, negative-sense RNA virus with a genome of approximately 19 kilobases in length that encodes for seven proteins: the surface glycoprotein (GP), nucleoprotein (NP), viral protein (VP) 35, VP40, VP30, VP24, and RNA-dependent RNA polymerase (L). The GP mediates host cell attachment and entry, while NP, VP35, VP30, and L, form the ribonucleoprotein complex responsible for genome transcription and replication. VP24 and VP40 function in virion assembly, packaging, and budding (Bodmer et al., 2024; Jain et al., 2021).

Few options exist for the prevention and treatment of EVD. The single-dose ERVEBO vaccine and the two-dose Zabdeno/Mvabea vaccine regimen are approved for prevention against Ebola virus, but not the other species in the genus (Kallay et al., 2024). The monoclonal antibodies Inmazeb and Ebanga, which target the Ebola virus glycoprotein are recommended for treatment (Taki et al., 2023). Although the nucleoside analog remdesivir showed potent anti-Ebola virus activity in cell culture and in nonhuman primates, remdesivir failed to demonstrate comparable therapeutic efficacy in human EVD patients (Mulangu et al., 2019; Warren et al., 2016, 2021). A related, orally bioavailable prodrug, obeldesivir, is effective against Ebola virus, Sudan virus, and Marburg virus in nonhuman primates, but its efficacy in humans remains unknown (Cross et al., 2024, 2025; Gilead, 2026; Woolsey et al., 2025). These limited prophylactic and therapeutic options underscore the need to broaden our treatment repertoire.

The Ebola virus L protein is the largest viral protein and possesses multiple enzymatic activities, including RNA-dependent RNA polymerase, methyltransferase, and guanylyltransferase functions (Jain et al., 2021; Valle et al., 2021). Ebola virus VP35 has been reported to have potential helicase activity despite not having a canonical helicase motif (Shu et al., 2019).

For many viruses, helicases are essential for key steps in the viral life cycle and represent attractive targets for antiviral drug development. The NS3 helicase of Hepatitis C virus, the NSP13 helicase of SARV-CoV-2, and the helicase-primase of Herpes Simplex Virus type 1 are essential for viral genome replication, remodeling, and packaging (Bermek and Williams, 2021; Grimes and Denison, 2024; Marecki et al., 2021). High-throughput drug screening efforts have identified compounds with potent activity against these viral helicases, including novel inhibitors and repurposed FDA-approved drugs (Otsuka et al., 2024; Salam and Akimitsu, 2013; Weller and Kuchta, 2013; Zeng et al., 2021).

Although Ebola virus lacks a canonical helicase motif, VP35 was previously reported to possess helicase-like activity (Shu et al., 2019). Based on this observation, we explored a library enriched for helicase-targeted small molecules as a phenotypic starting point for anti-Ebola drug discovery without assuming that VP35 was necessarily the molecular target. Therefore, we screened a library of 2620 small molecules with annotated activity against cellular and viral helicases against a reporter Ebola virus. The screen was performed using an established biologically contained Ebola virus that lacks the VP30 gene (hereafter referred to as Ebola∆VP30 virus) in Vero cells stably expressing VP30 (Vero VP30 cells). This platform enables the safe evaluation of compounds against Ebola virus in a biosafety level 2 setting (Halfmann et al., 2008). Seven compounds significantly reduced virus titers at least 100-fold with minimal cytotoxicity with two compounds completely inhibiting Ebola∆VP30 virus infection. Mechanistic studies suggested that the compounds may target glycoprotein-mediated virus entry, a viral or cellular component involved in viral RNA synthesis, or another, as yet undefined, antiviral mechanism.

## 2 Materials and methods

### 2.1 The EbolaΔVP30 virus system and cell lines

A biologically contained Ebola virus (Halfmann et al., 2008) expressing the Renilla luciferase gene (Ebola∆VP30 RenLuc virus) was used in these studies. The use of this virus system is approved for biosafety level 2 (BSL-2) containment at the University of Wisconsin by the Institutional Biosafety Committee, the National Institutes of Health, and the Centers for Disease Control and Prevention.

Vero E6 cells that stably express Ebola VP30 (Vero VP30) and wild-type Vero E6 cells were maintained in Minimum Essential Medium (MEM) supplemented with 10% fetal calf serum (FCS), antibiotics, and 5 µg/ml of puromycin (Vero VP30 cells only). HEK 293T cells were maintained in Dulbecco’s Modified Eagle Medium (DMEM) supplemented with 10% FCS and antibiotics. All cell lines were maintained at 37 °C and 5% CO_2_ and were routinely tested for mycoplasma.

### 2.2 Library screen

We screened a commercially available library of 2620 small molecules annotated for activity against cellular and viral helicases (Life Chemicals) to identify phenotypic antiviral hits. These compounds were evaluated for antiviral activity and cytotoxicity on Vero VP30 cells.

For the initial assessment, compounds were prepared at a concertation of 20 µM by diluting the 10 mM stock in infection medium (MEM supplemented with 2% FCS and antibiotics). Then, 50 uL of diluted compound or DMSO control (prepared using the same dilution scheme to ensure an equivalent final DMSO concentration) was added to Vero VP30 cells, which were seeded in 96-well plates (white-walled, clear bottom; Corning) the day prior. After an incubation of 1 h at 37 °C, 50 µL of Ebola∆VP30 RenLuc virus at an multiplicity of infection (MOI) of 0.01 was added to the cells resulting in a final concentration of 10 µM for each compound. After 48 h, 100 µL of EnduRen Live Cell Substrate (Promega) reconstituted in DMSO was mixed with 50 mL of medium and 100 uL/well was added to the wells. Luciferase activity was measured by reading luminescence 24 h later. Cell viability was then assessed using an MTS-based assay by adding 20 µL/well of CellTiter 96 AQ_ueous_ One Solution reagent (Promega) to the wells. After 4 h, absorbance was measured at a wavelength of at 490 nm on a Tecan M1000 plate reader. Percent infection and viability of cells treated with compounds was then compared to DMSO-treated cells. Compounds that exhibited efficient antiviral activity (infection reduced by ≥ 80%) with low cytotoxicity (≥ 90% cell viability) were selected for further evaluation.

### 2.3 Virus infection and plaque assay

Vero VP30 cells seeded one day prior in TC24 plates were incubated for 1 h at 37 °C with 500 µL of 5 µM compound or an equal volume of DMSO diluted in infection medium. Medium was then aspirated and 100 µL of Ebola∆VP30 RenLuc virus at an MOI of 0.001 was added to the wells. Cells were then incubated at 37 °C for 1 h with rocking every 15 min, after which the cells were washed three times with infection medium, and then 500 µL of infection medium with either 5 µM compound or DMSO (equal volume as the compound) was added back to the cells. After 4 days, 200 µL of cell culture supernatant was collected from each well and frozen in −80 °C. Each compound was tested in triplicate.

For virus titration, samples were serially diluted 10-fold from 10^–1^ to 10^–5^ in infection medium. The original medium was aspirated from Vero VP30 cells seeded a day prior in TC24 plates and 100 µL of diluted virus added, followed by incubation at 37 °C for 1 h, with rocking every 15 min. Cells were then washed three times with infection medium and then 1 ml of 1.25% methyl cellulose medium (MCM) supplemented with 2% FCS was added and incubated at 37 °C. After 7 days, the cells were fixed with 10% formalin for at least 2 h, washed five times with PBS, treated with 0.25% Triton X-100 in PBS for 15 min, and then washed four times with PBS. A mouse anti-VP40 (Halfmann et al., 2008) primary antibody (1:10,000 dilution in PBS) was then added and the plates incubated at 4 °C overnight. The next day, the plates were washed four times with PBS and then incubated with goat anti-mouse IgG–horseradish peroxidase-conjugated secondary antibody (1:1000 dilution; Invitrogen) for 1 h at room temperature. After the secondary antibody was washed off with PBS, 3,3′-diaminobenzidine (DAB) solution (Sigma) was added to stain for viral plaques, with staining terminated after a few minutes by quenching with water.

### 2.4 Antiviral potency and cytotoxicity assays

Half-maximal inhibitory concentration (IC_50_) values were determined by serially diluting compounds from 50 µM to 0.1 µM in infection medium. Vero VP30 cells seeded the day prior in 96-well plates (Corning) were pre-treated with 50 µL of diluted compounds for 1 h at 37 °C. Ebola∆VP30 RenLuc virus was added to each well at an MOI of 0.01 in a 50-µL volume, and the plates were incubated at 37 °C for 72 h. Virus-driven luciferase activity was measured using ViviRen Live Cell substrate (Promega) by adding 100 µL of ViviRen Live Cell substrate that was mixed with 100 ml of infection medium; 50 µL of diluted substrate was added to each well and luminescence was measured on a Tecan M1000 plate reader.

Cytotoxic concentration 50% (CC_50_) values were determined by serially diluting compounds from 100 µM to 0.2 µM and adding 50 µL of serially diluted compounds to Vero VP30 cells seeded in 96-well plates the day prior. After an incubation at 37 °C for 72 h, RealTime-Glo MT Cell Viability reagent (Promega) diluted in infection medium at a ratio of 1:2000, was add at 100 µL/well and luminescence was measured on a Tecan M1000 plate reader. For both IC_50_ and CC_50_ assays, DMSO controls were prepared using the same dilution scheme as the compounds to ensure equivalent DMSO concentrations. The selective index (SI) of each compound was calculated by dividing the CC_50_ value by the IC_50_ value.

### 2.5 Mechanism of action analyzes

#### 2.5.1 Minigenome assay

To evaluate viral replication and transcription, HEK 293T cells were transfected using LT-1 (Mirus) and the following plasmids: pCAGGS plasmids expressing the Ebola virus replication proteins, VP30 (0.1 µg/well), NP-VP35 (0.05 µg/well), L (0.3 µg/well), the RNA T7 polymerase to generate virus-like RNA (0.1 µg/well), the minigenome plasmid, p3E5E containing 3′ leader and 5′ trailer sequences of the Ebola virus genome flanking the firefly luciferase reporter gene (p3E5E-FFLuc; 0.1 µg/well), and pCAGGS Renilla luciferase (0.002 µg/well) as an internal control. To determine the background levels of firefly luciferase in the minigenome assay, the Ebola L plasmid was replaced with pCAGGS expressing GFP (0.3 µg/well). Six hours after transfection, compounds or an equal volume of DMSO were added to the cells at a final concentration of 5 µM. At 48 h after transfection, luciferase values were measured using substrates in the dual-luciferase reporter assay kit (Promega) according to the manufacturer’s instructions. Firefly luciferase activity, reflecting Ebola virus minigenome replication and transcription, was normalized to Renilla luciferase activity to account for differences in transfection efficiency and cell number. Normalized values were expressed relative to the DMSO-treated control, which was set to 100% with each compound tested in duplicate.

#### 2.5.2 Entry inhibition

To evaluate virus entry, pseudotyped replication-competent vesicular stomatitis virus (VSV) expressing the glycoprotein of Ebola (VSV-EBOV GP), the glycoprotein of the closely related Marburg virus (VSV-MARV GP) or the glycoprotein (GPC) of an unrelated virus, Andes virus, a New World hantavirus (VSV-Andes GPC) were used. Wild-type Vero cells, seeded in TC24 well plates the day prior, were treated with 5 µM compound or an equal volume of DMSO for 1 h. Cells were then infected with VSV-EBOV GP, VSV-MARV GP, or VSV-Andes GPC at an MOI of 0.001 for 30 min, with rocking every 15 min. Unbound virus was then washed off twice and normal growth medium containing compound at 5 µM or DMSO was added back to the cells. Two days after infection, cell culture supernatants were collected, and plaque assays were performed on wild-type Vero cells overlayed with 0.75% MCM supplemented with 2% FCS and antibiotics.

### 2.6 Software and figures

Dose-response curves were fitted nonlinear regression in GraphPad Prims 9 software. Statistical analysis was performed using GraphPad Prism 9. Dunnett’s multiple comparisons test was used to calculate the statistical significance indicated throughout the manuscript and in the figure legends. BioRender was used to aid in the generation of Fig. 5 while GraphPad Prism 9 was used to generate the figures in Fig. 1–4.

**Fig. 1:**
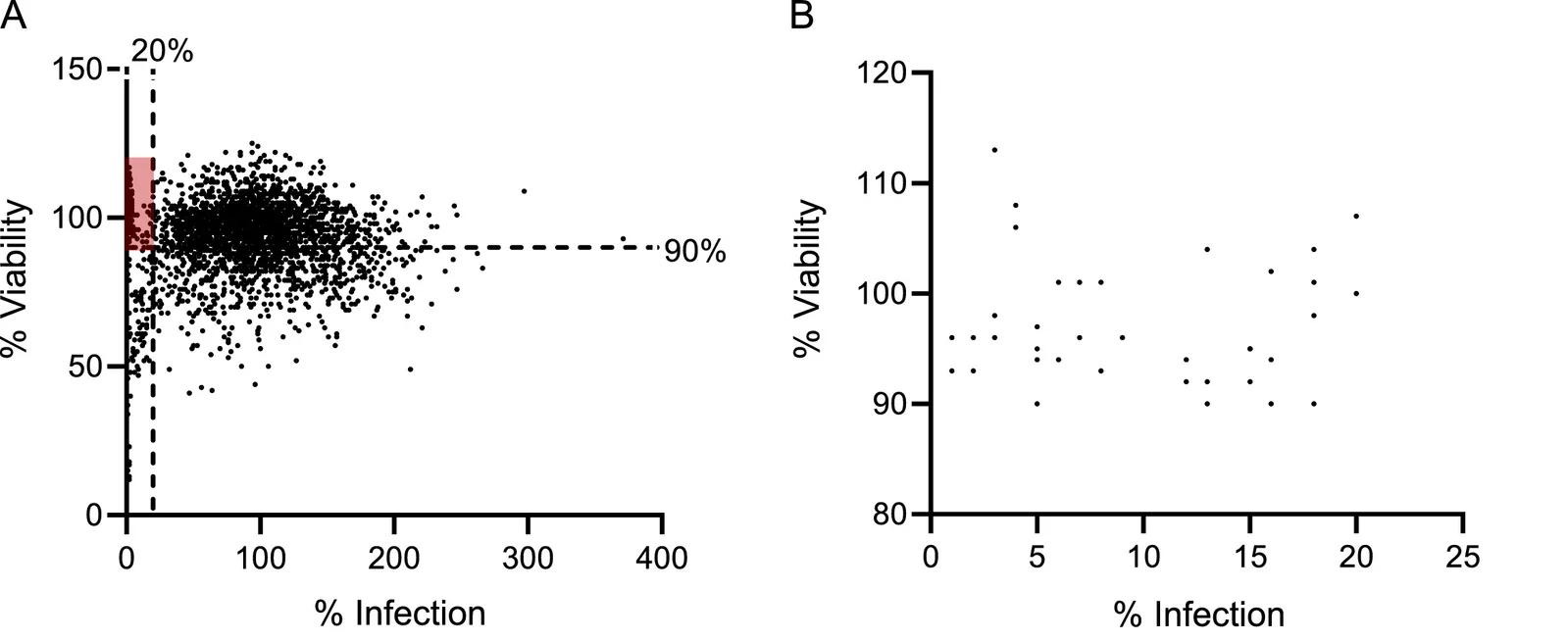
**Phenotypic screening of a helicase-annotated chemical library against EbolaΔVP30-RenLuc virus.** (A). A library of 2620 compounds annotated for cellular or viral helicase inhibitory activity were screen in a phenotypic infection assay against Ebola∆VP30 RenLuc virus. Vero VP30 cells were pre-treated with each compound at a concentration of 10 µM for 1 h, then infected with Ebola∆VP30 RenLuc virus at an MOI of 0.01. Luminescence was measured 72 h post-infection to quantify virus replication, and cell viability was assessed in parallel using an MTS-based assay. Percent infection and viability were normalized to a DMSO-treated control. The red boxed region denotes compounds with notable antiviral activity and minimal cytotoxicity. (B) Enlarged insert of the red boxed region in Fig. 1A, highlighting the 36 compounds that reduced Ebola∆VP30 RenLuc virus infection by ≥ 80% while maintaining ≥ 90% cell viability.

## 3 Results

### 3.1 Identifying compounds with antiviral activity against EbolaΔVP30 virus

To identify anti-Ebola chemical scaffolds, we screened a library of 2620 compounds annotated for helicase inhibitory activity using a cell-based phenotypic assay using EbolaΔVP30 virus. Of these compounds screened, most (1837 compounds, 70% of the library compounds) were not cytotoxic, maintaining > 90% cell viability on Vero VP30 cells compared to DMSO-treated cells (Fig. 1A). From the screen, we identified 183 compounds (7% of the library compounds) that reduced Ebola∆VP30 virus replication by ≥ 80%, as measured by Renilla luciferase expression, 36 of which showed low cytotoxicity (≥ 90% cell viability) (Fig. 1B).

### 3.2 Validation of antiviral activity at a single time point

Based on our initial results, 32 compounds from the screen (four compounds were not available for individual purchase) that showed antiviral activity with minimal cytotoxicity were selected for a validation study. Vero VP30 cells were treated with DMSO or each compound at a concentration of 5 µM for 1 h and then infected with Ebola∆VP30 RenLuc virus at an MOI of 0.001. After infection, any unbound virus was washed off and medium with DMSO or compound at 5 µM was added back to the cells. Four days after infection, cell culture supernatants were collected, and viral titers were determined by plaque assays on Vero VP30 cells.

Of the 32 compounds tested, 15 showed no to minimal (< 10-fold) reductions in viral titers (Fig. 2, white bars). Six compounds reduced titers by 10- to 100-fold (blue bars), whereas 11 compounds (orange bars) reduced titers by >100-fold with statistically significant reduction for seven of these 11 compounds. Two compounds (F2019–2014 and F2019–2141) completely inhibited Ebola∆VP30 RenLuc virus replication.

**Fig. 2:**
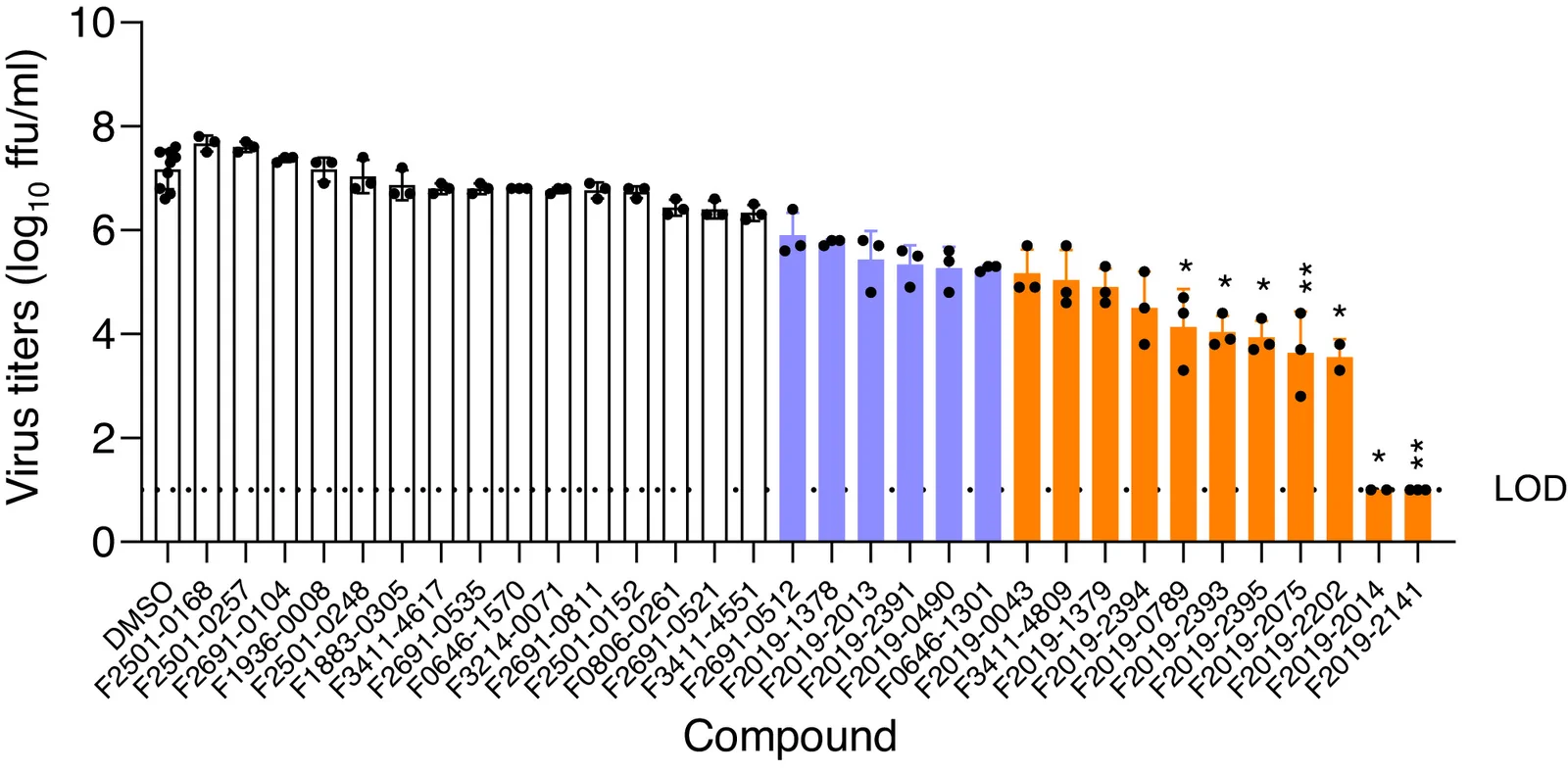
**Reduction in EbolaΔVP30 RenLuc virus titers following treatment with selected compounds.** Vero VP30 cells were treated with compounds at a concentration of 5 µM for 1 h before being infected with virus at an MOI of 0.001. Unbound virus was then washed off and DMSO or compound (5 µM concentration) was added back to the cells. Four days after infection, cell culture supernatants were collected, and virus titers were determined by performing plaque assays on Vero VP30 cells. Data are from a single experiment with two or three independently treated wells for each compound. DMSO-treated control wells were included on multiple plates. Statistical analysis was determined using one-way ANOVA followed by Dunnett’s multiple-comparisons test, comparing each compound with the DMSO-treated control samples. * *p* < 0.05: ** *p* < 0.01. All other values were not statistically significant. Focus-forming units, ffu; LOD, limit of detection.

### 3.3 Cell viability and antiviral potency

The IC_50_ and CC_50_ values of the 11 compounds that reduced virus titers by >100-fold were determined on Vero VP30 cells. All compounds had CC_50_ values of 14.9 µM or greater, with F2019–0043 demonstrating no toxicity up to 100 µM in the assay (Table 1). The IC_50_ values of the 11 compounds ranged from 1.6 to 5.6 µM. Compounds F2019–2014 and F2019–2141, which completely inhibited virus replication, demonstrated the best antiviral potencies with IC_50_ values of 1.7 µM and 1.9 µM, respectively.

**Table 1:** Half-maximal inhibitory concentration (IC_50_), 50% cytotoxic concentration (CC_50_), and selectivity indices (SI) of the 11 compounds that reduced virus titers by >100-fold.

Compound ID	IC_50_ (µM)	CC_50_ (µM)	SI (CC_50_/IC_50_)
F2019–0043	2.2	>100	47.3
F3411–4809	3.0	20.8	6.8
F2019–1379	5.6	33.8	6.0
F2019–2394	2.0	30.4	15.4
F2019–0789	1.8	34.2	18.9
F2019–2393	4.5	16.8	3.7
F2019–2395	1.6	14.9	9.2
F2019–2075	2.4	21.4	8.7
F2019–2202	4.4	24.1	5.4
F2019–2014	1.7	14.9	9.0
F2019–2141	1.9	19.4	10.4

### 3.4 Initial mechanism of action studies

To begin to understand the mechanism of action of the 11 compounds (Supplementary Table 1) that reduced virus titers by at least 100-fold, we first determined whether any of the compounds could interfere with viral replication/transcription using an Ebola virus minigenome assay (Jasenosky et al., 2010). Of the 11 compounds, five had minimal impact on minigenome activity, reducing normalized minigenome firefly luciferase expression by 3%–15% compared to DMSO-treated cells (Fig. 3A). The remaining six compounds (F3411–4809, F2019–1379, F2019–2393, F2019–2395, F2019–2075, and F2019–2141) significantly reduced minigenome activity. Among these, F2019–2141, one of the two compounds that completely inhibited EbolaΔVP30 virus replication, exhibited the greatest reduction (50%) compared to DMSO-treated cells (Fig. 3A). These findings suggest that some compounds may affect viral RNA synthesis, either directly or indirectly through cellular processes required for minigenome activity.

**Fig. 3:**
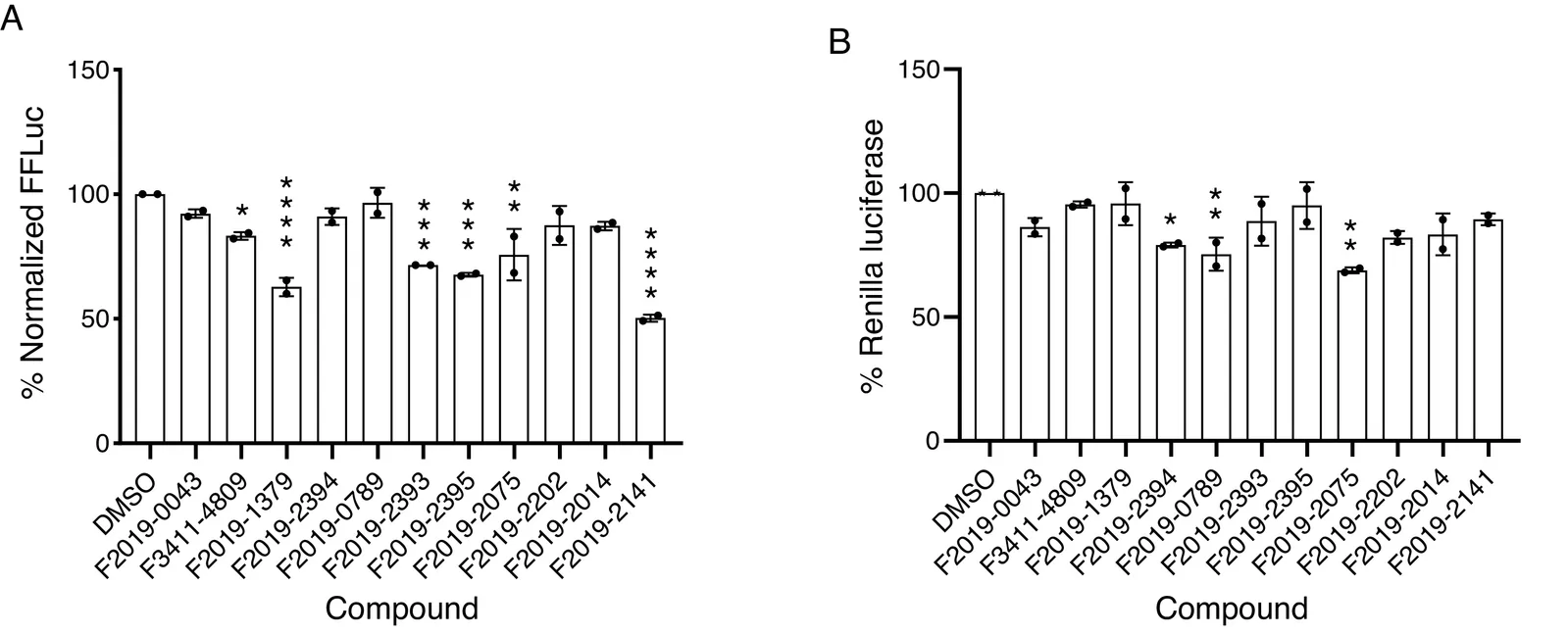
**Mechanistic effect of compounds on Ebola virus replication/transcription in a minigenome assay**. The 293T cells were transfected with pCAGGS expression plasmids encoding the L, NP-VP35, VP30, T7 polymerase, the minigenome plasmid p3E5E-fLuc, and the transfection efficiency control plasmid pRenLuc. Six hours later, they were treated with 5 µM compounds or an equal volume of DMSO. Forty-eight hours leter, the cells were lysed, and a dual-Luciferase reporter assay was used to quantify Firefly luciferase and Renilla luciferase activity by reading luminescence. (A) Minigenome activity as a ratio of Firefly luciferase/Renilla luciferase of cells treated with compounds or DMSO. (B) Toxicity of compounds represented by percent Renilla luciferase activity of cells treated with compounds vs. DMSO. Data are from a single experiment with two independently treated wells for each compound. Statistical analysis was performed using a one-way ANOVA followed by Dunnett’s multiple-comparisons test comparing each compound with DMOS treated controls. * *p* < 0.05: ** *p* < 0.01: *** *p* < 0.001: **** *p* < 0.0001. All other values were not statistically significant. FFLuc, firefly luciferase.

To assess potential nonspecific effects, we measured Renilla luciferase expression driven by a cellular promoter using a pCAGGS plasmid. Three compounds significantly reduced Renilla luciferase levels, with F2019–2075 showing significant inhibition of both Ebola minigenome-driven and host-driven luciferase expression suggesting potential general cytotoxicity or off-target effects rather than specific interference with the viral replication/transcription in the minigenome assay (Fig. 3B). These results suggest that the effects of some compounds on minigenome activity may, at least in part, reflect broader effects on cellular processes rather than specific inhibition of viral replication/transcription.

Next, we evaluated whether any of the compounds inhibited viral glycoprotein-dependent entry by using a replication competent vesicular stomatitis virus (VSV) expressing the Ebola GP (VSV-Ebola GP), Marburg GP (VSV-Marburg GP) or VSV-Andes GPC. The latter served as a control to distinguish Ebola and Marburg virus GP-specific inhibition of viral entry from nonspecific inhibition of post-entry VSV replication. Six compounds significantly reduced VSV-Ebola GP virus titers by at least 100-fold compared to DMSO (Fig. 4). Two of these compounds (F2019–0789 and F2019–2075) were specific to VSV-Ebola GP and did not significantly inhibit VSV entry mediated by the Marburg GP or Andes GPC. Four compounds (F2019–2393, F2019–2202, F2019–2014, and F2019–2141) significantly inhibited both VSV-Ebola GP and VSV-Marburg GP, but not VSV-Andes GPC. Among these, F2019–2014 was one of the two compounds that completely inhibited EbolaΔVP30 virus replication. F3411–4809 completely inhibited entry mediated by the Andes GPC but had no effect on VSV-Ebola GP or VSV-Marburg GP.

**Fig. 4:**
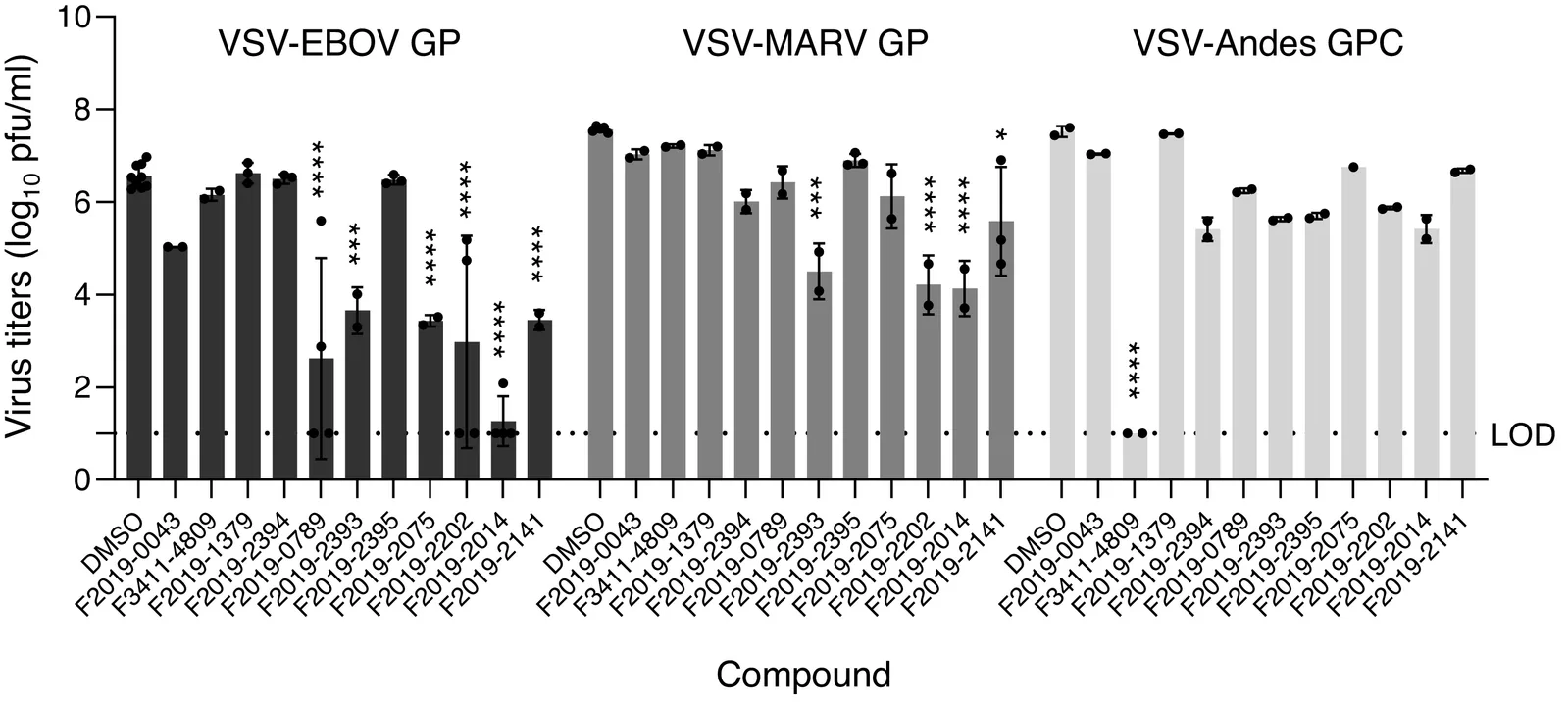
**Mechanistic effects of compounds on virus entry.** Entry inhibition assay showing the effect of compounds on infection of wild-type Vero cells by vesicular stomatitis virus (VSV) expressing different viral glycoproteins. Vero cells, pre-treated for 1 h with 5 µM compounds or an equal volume of DMSO, were infected with VSV expressing Ebola GP (VSV-Ebola GP), Marburg GP (VSV-Marburg GP), or Andes virus GPC (VSV-Andes GPC) at an MOI of 0.001. Cell culture supernatants were collected at 48 h, titrated, and plaque assayed to quantify viral titers. Data are from a single experiment with two or three independently treated wells for each compound. DMSO-treated control wells were included on multiple plates. Statistical analysis was performed using a one-way ANOVA followed by Dunnett’s multiple-comparisons test comparing each compound with DMOS treated controls. * *p* < 0.05: ** *p* < 0.01: *** *p* < 0.001: **** *p* < 0.0001. All other values were not statistically significant. Plaque-forming units, pfu; LOD, limit of detection.

## 4 Discussion

We screened a library of 2620 small molecules from a helicase-annotated library to identify inhibitors of Ebola virus infection in a cell-based phenotypic infection assay using a live EbolaΔVP30 reporter virus assay. Because no robust, reproducible biochemical assay is available to directly measure the putative helicase activity of Ebola VP35, compounds were evaluated in a phenotypic infection assay rather than selected based on inhibition of VP35 enzymatic activity. We identified 11 compounds that effectively reduced Ebola virus infection with low cytotoxicity in Vero VP30 cells, including two compounds that completely inhibited EbolaΔVP30 virus infection. Our findings suggestion that these compounds may exert their antiviral effects with varying degrees of efficacy and through different mechanisms (Fig. 5). F2019–2141 was of particular interest because of its ability to completely inhibit EbolaΔVP30 virus replication, reduce Ebola virus minigenome activity by 50% and significantly inhibit VSV infection mediated by Ebola virus GP and Marburg virus GP. Although these findings raise the possibility of nonspecific inhibitory effects, the compound did not affect luciferase expression driven by a cellular promoter or VSV infection mediated by the Andes virus GPC, suggesting some degree of specificity in its antiviral activity. F2019–2014 exhibited a similar antiviral profile but did not significantly inhibit Ebola virus minigenome activity. In contrast, F2019–2394 and F2019–0043 reduced EbolaΔVP30 virus titers by at least 100-fold but exhibited minimal inhibitory activity in the VSV infection and minigenome assays.

**Fig. 5:**
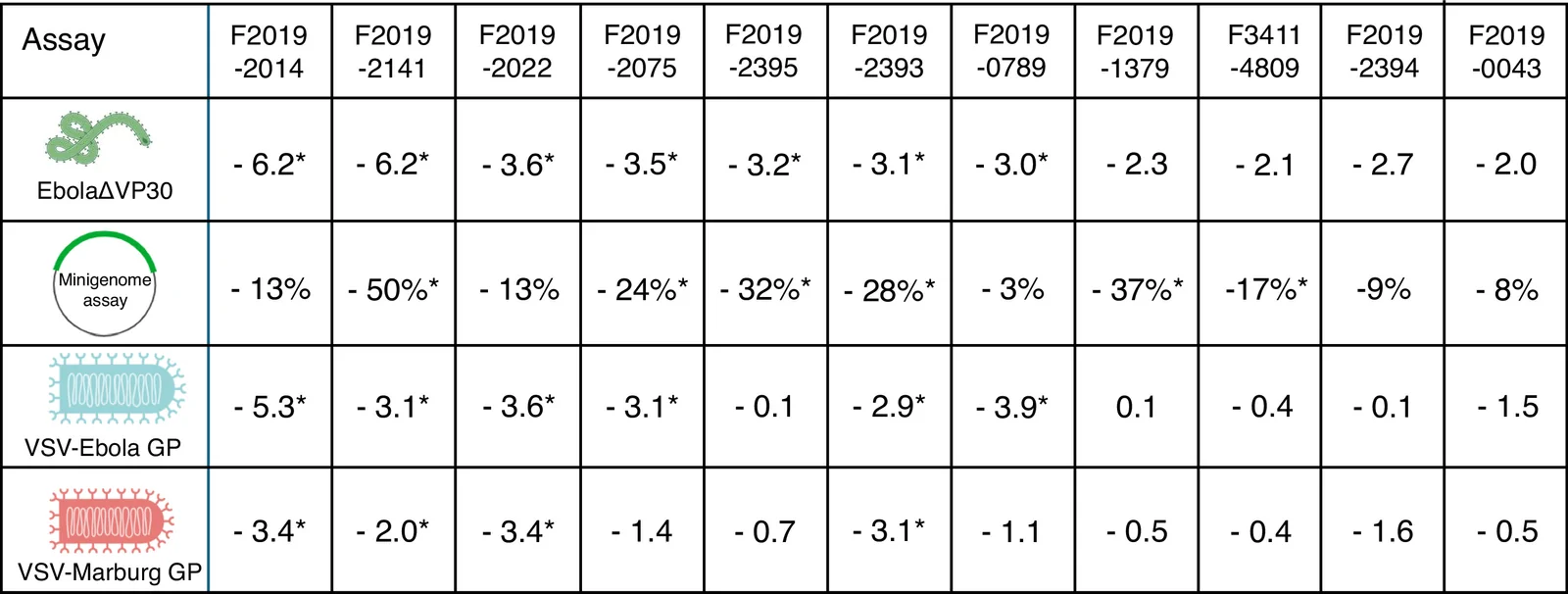
**Summary of antiviral activity.** The reduction of EbolaΔVP30 virus titers and the extent of inhibition observed in subsequent assays assessing glycoprotein-dependent infection and Ebola virus minigenome activity are summarized for each compound. A minus sign (–) indicates a reduction in the respective assay relative to the DMSO-treated control, and an asterisk (*) denotes statistically significant inhibition.

Viral helicases are attractive antiviral targets because they play key roles in the viral life cycle, including remodeling RNA-RNA and RNA-protein complexes during genome replication and transcription, while also contributing to evasion of host innate immune responses (Marecki et al., 2021). Many RNA and DNA viruses encode dedicated helicase proteins, such as members of the Coronaviridae, whereas others contain helicase domains within multifunctional proteins, as seen in the Herpesviridae. In contrast, some viruses, including members of the Retroviridae, rely on host helicases to support their replication (Heaton et al., 2023). Although Ebola virus does not encode a canonical helicase, VP35 has been reported by one group to possess helicase-like activity despite lacking conserved helicase motifs (Shu et al., 2019), although there have been no additional reports on its activity. Nevertheless, this observation provided the rationale for screening a library enriched for compounds with reported helicase-related activity against Ebola virus infection.

An important limitation of our study is that the molecular targets and more detailed mechanisms of action of the identified compounds remain to be determined. Because the putative helicase activity of Ebola virus VP35 cannot be measured directly, we were unable to determine whether compounds that inhibited in the Ebola minigenome assay did so by directly targeting the helicase-like activity of VP35. Nevertheless, our findings demonstrate that compounds selected from a library enriched for reported helicase-related activity can effectively inhibit Ebola virus infection and provide a strong foundation for future mechanistic studies.

Although additional studies are required to define the molecular targets and optimize antiviral activity, the compounds identified in this study establish new chemical scaffolds for anti-filovirus drug discovery. Given their modest selectivity indices (SI = 3.7– 47.3; Table 1), these scaffolds will require extensive medicinal chemistry optimization to improve selectivity and expand their therapeutic windows before advancing to in vivo evaluation. Nevertheless, these preliminary screening hits represent starting points for the development of broad-spectrum therapeutics effective against Ebola and Marburg viruses, addressing an unmet need in filovirus outbreaks.

## Funding source

This work was supported by the National Institutes of Health AViDD U19 AI171292; University of North Carolina University “Rapidly Emerging Antiviral Drug Discovery AViDD Center (READDI-AC)”.

## CRediT authorship contribution statement

**Meareg G. Amare:** Writing – original draft, Methodology, Investigation, Formal analysis. **Peter J. Halfmann:** Writing – review & editing, Supervision, Funding acquisition, Conceptualization.

## Declaration of competing interest

The authors declare that they have no known competing financial interests or personal relationships that could have appeared to influence the work reported in this paper.

## Data Availability

The data that generated the figures in this paper can be found in the Source File.
