# Supplementary material for: Identification of small molecule inhibitors of Ebola virus by use of a biologically contained virus system
Source: Virus Res. 2026 Oct 25;373:199823. doi: 10.1016/j.virusres.2026.199823 (PMC13734852; doi:10.1016/j.virusres.2026.199823)
Supplement: Supplementary file 1 [file mmc2.pdf]

Supplementary Table 1: Information of the 11 compounds that reduced virus titers by more than 100-fold, including their molecular formulas, molecular weights, purity and CAS Registry Numbers.

Compound ID	Formula	Molecular weight (g/mol)	Purity (%)	CAS Registry Number
F2019-0043	$C_{26}H_{35}ClN_4O_3S_2$	514.70	90	1135235-47-0
F3411-4809	$C_{27}H_{33}ClN_4OS$	497.10	90	1189446-59-0
F2019-1379	$C_{28}H_{32}ClFN_4O_3S_2$	554.70	90	1135139-65-9
F2019-2394	$C_{29}H_{35}ClN_4O_3S_2$	550.74	90	1135222-78-4
F2019-0789	$C_{26}H_{35}ClN_4O_3S_2$	514.70	90	1216553-03-5
F2019-2393	$C_{28}H_{39}ClN_4O_3S_2$	542.76	90	1135221-58-7
F2019-2395	$C_{30}H_{37}ClN_4O_3S_2$	564.76	90	1135223-11-8
F2019-2075	$C_{27}H_{37}ClN_4O_3S_2$	528.73	90	1135198-33-2
F2019-2202	$C_{27}H_{37}ClN_4O_3S_2$	528.73	90	1135199-87-9
F2019-2014	$C_{29}H_{35}ClN_4O_4S_2$	566.73	90	1135132-92-1
F2019-2141	$C_{30}H_{37}ClN_4O_3S_2$	564.76	90	1135211-28-7
